# Toll-like receptor 2 activation depends on lipopeptide shedding by bacterial surfactants

**DOI:** 10.1038/ncomms12304

**Published:** 2016-07-29

**Authors:** Dennis Hanzelmann, Hwang-Soo Joo, Mirita Franz-Wachtel, Tobias Hertlein, Stefan Stevanovic, Boris Macek, Christiane Wolz, Friedrich Götz, Michael Otto, Dorothee Kretschmer, Andreas Peschel

**Affiliations:** 1 Department of Infection Biology, Interfaculty Institute for Microbiology and Infection Medicine Tübingen (IMIT), University of Tübingen, Auf der Morgenstelle 28, 72076 Tübingen, Germany; 2 Pathogen Molecular Genetics Section, Laboratory of Bacteriology, National Institute of Allergy and Infectious Diseases, National Institutes of Health, Bethesda, Maryland 20892, USA; 3 Proteome Center Tübingen, Interfaculty Institute of Cell Biology, University of Tübingen, Auf der Morgenstelle 15, 72076 Tübingen, Germany; 4 Institute for Molecular Infection Biology, University of Würzburg, Josef-Schneider-Straße 2, Würzburg 97080, Germany; 5 Department of Immunology, Interfaculty Institute for Cell Biology, Eberhard Karls University, Auf der Morgenstelle 15, 72076 Tübingen, Germany; 6 Department of Medical Microbiology and Hygiene, Interfaculty Institute for Microbiology and Infection Medicine Tübingen (IMIT), University of Tübingen, Elfriede-Aulhorn-Straße 5, 72076 Tübingen, Germany; 7 Department of Microbial Genetics, Interfaculty Institute for Microbiology and Infection Medicine Tübingen (IMIT), University of Tübingen, Auf der Morgenstelle 28, 72076 Tübingen, Germany; 8 German Center for Infection Research, University of Tübingen, Auf der Morgenstelle 28, 72076 Tübingen, Germany

## Abstract

Sepsis caused by Gram-positive bacterial pathogens is a major fatal disease but its molecular basis remains elusive. Toll-like receptor 2 (TLR2) has been implicated in the orchestration of inflammation and sepsis but its role appears to vary for different pathogen species and clones. Accordingly, *Staphylococcus aureus* clinical isolates differ substantially in their capacity to activate TLR2. Here we show that strong TLR2 stimulation depends on high-level production of phenol-soluble modulin (PSM) peptides in response to the global virulence activator Agr. PSMs are required for mobilizing lipoproteins, the TLR2 agonists, from the staphylococcal cytoplasmic membrane. Notably, the course of sepsis caused by PSM-deficient *S. aureus* is similar in wild-type and TLR2-deficient mice, but TLR2 is required for protection of mice against PSM-producing *S. aureus*. Thus, a crucial role of TLR2 depends on agonist release by bacterial surfactants. Modulation of this process may lead to new therapeutic strategies against Gram-positive infections.

## Abstract (web summary)

The role played by human protein TLR2 in inflammation and sepsis varies for different bacterial pathogens. Here, Hanzelmann *et al*. show that the differential abilities of *Staphylococcus aureus* strains to activate TLR2 depend on their production of peptides that release lipoproteins known to act as TLR2 agonists.

## Graphical abstract

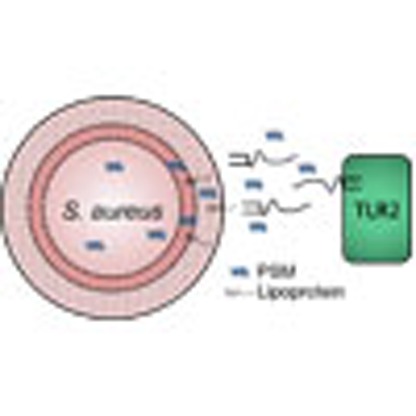

Sepsis is a severe syndrome of increasing frequency with a very high mortality rate^1^. Systemic inflammation initiated by conserved microbe-associated molecular pattern (MAMP) molecules from circulating bacteria can lead to septic shock and organ failure. Toll-like receptor 4 (TLR4) and lipopolysaccharide, its microbial agonist, are long known as major factors in infections and sepsis caused by Gram-negative bacteria^2^,^3^. In contrast, TLR2 activates immune cells in response to bacterial lipoproteins and has been considered as a major factor in Gram-positive sepsis^4^,^5^. Certain alleles in the TLR2 pathway associate with increased susceptibility and severity of sepsis caused by major Gram-positive pathogens such as *Staphylococcus aureus*^6^,^7^. However, the role of TLR2 in sepsis is much more elusive than that of TLR4 because TLR2 appears to have ambivalent roles and its importance seems to vary for different pathogens for unknown reasons^8^,^9^,^10^. In particular, it has remained elusive under which conditions TLR2 may affect the course of infection and elicit protective or even detrimental responses^5^,^11^,^12^. These unsolved questions have impeded the use of TLR2 as a target for immunomodulatory drugs^12^,^13^.

A major percentage of bacteremia and sepsis cases is caused by *S. aureus* and these infections are often particularly difficult to treat because of the enormous burden of antibiotic resistance in methicillin-resistant *S. aureus* (MRSA)^14^. The extraordinary capacity of *S. aureus* to cause massive inflammation and withstand host defense can be attributed to the release of pro-inflammatory MAMPs and leukocyte-damaging toxins^15^,^16^. TLR2-activating lipoproteins, major staphylococcal MAMPs, are attached to bacterial membranes via a diacylglycerol moiety, which is connected to an N-terminal cysteine residue of the lipoprotein via a thioether bond by the lipoprotein diacylglyceryl transferase (Lgt)^17^. In addition, many bacteria modify the lipoprotein *N*-terminus with a third fatty acid^18^. Many lipoproteins from Gram-positive bacteria are components of ABC importers such as the staphylococcal siderophor uptake system SitC and are upregulated under nutrient-limited conditions^19^,^20^. Inactivation of *lgt* abrogates the post-translational modification of pre-lipoproteins and the capacity of *S. aureus* to stimulate TLR2^21^. In addition to MAMPs a large inventory of toxins compromizing the function of leukocytes or other host cells contribute to the aggressive behaviour of *S. aureus*^22^.

Most of the *S. aureus* virulence factors are controlled by the quorum-sensing regulatory Agr system, which is specific for staphylococci^23^. AgrA, the DNA-binding regulator of the Agr system controls directly expression of phenol-soluble modulin (PSM) peptides, which shape *S. aureus* infections in several ways^24^,^25^. *S. aureus* usually produces four co-transcribed short PSMα peptides (20–22 amino acids), two longer PSMβ peptides, and the PSMα-related δ-toxin, which is encoded in RNAIII of the Agr system^26^. PSMs are α-helical and have surfactant-like properties, which are responsible for the potent cytolytic activity of PSMs towards leukocytes and other host cells^24^. Whereas this activity depends on high (micromolar) concentrations PSMs can stimulate and activate leukocytes in the nanomolar range because they bind to the G-protein coupled formylpeptide receptor 2 (FPR2) thereby representing chemotactic MAMPs^27^,^28^. Staphylococcal species and strains differ largely in the levels of PSM expression, which appears to affect the virulence of the producing strain^29^. However, how important PSMs may be for the induction of inflammation in the context of other MAMPs such as lipoproteins has remained unknown.

Here we analyse how staphylococcal strains differ in their capacity to activate TLR2 and report that PSMs are crucial for the proinflammatory potential of *S. aureus* because they are required for the release of lipoproteins from bacterial cytoplasmic membranes. Accordingly, PSMs have a strong impact on the severity of sepsis, and TLR2 has an important role in systemic *S. aureus* infections only for PSM-producing strains.

## Results

### TLR2 activation depends on an active Agr system

**Figure 1:**
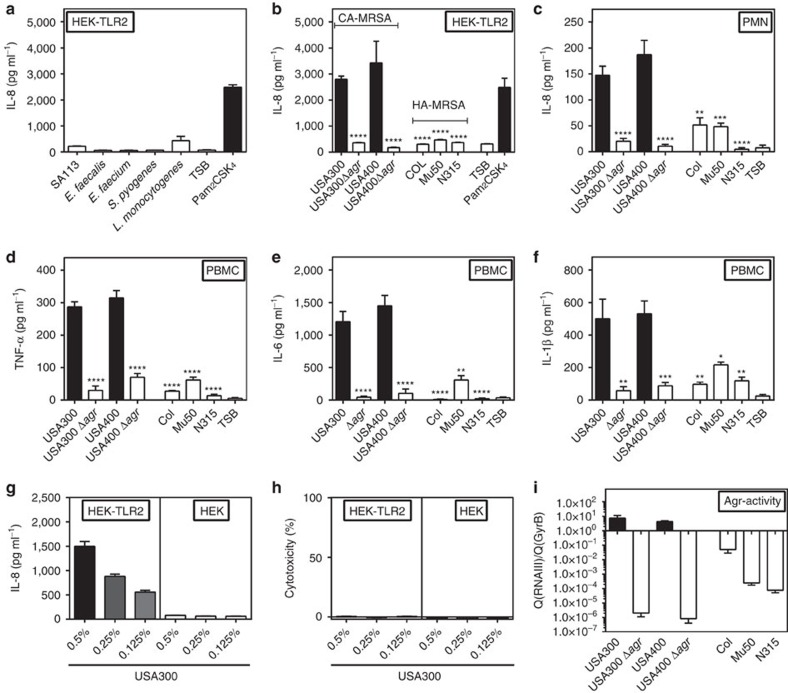
The *S. aureus* master regulator of virulence Agr governs the capacity of *S. aureus* to stimulate TLR2. Culture filtrates of *S. aureus* strains with strong Agr activity but not with weakly active or no Agr have potent capacities to induce either IL-8 in HEK-TLR2 cells (**a**,**b**) and in PMN (**c**), or TNF-α, IL-6 and IL-1β in PBMCs (**d**–**f**). The synthetic lipopeptide Pam_2_CSK_4_ and TSB medium were used as positive and negative controls, respectively. Expression of TLR2 in HEK cells leads to dose-dependent IL-8 induction by USA300 culture filtrates (**g**). Culture filtrates are not cytotoxic to HEK cells at indicated dilutions used for stimulation (**h**). Agr activity of *S. aureus* strains was measured by qRT–PCR of RNAIII expression (**i**). Data represent means +/− s.e.m. of at least three independent experiments. *P<0.05, **P<0.01, ***P<0.001; ****P<0.0001 significantly different versus USA300 wild-type (**b**–**f**) as calculated by Student's *t* test.

*S. aureus* strains differ in their ability to stimulate TLR2. Some strains are almost unable to trigger TLR2 even in the presence of lipoproteins suggesting that other factors besides lipoproteins must have a role^5^,^11^. Accordingly, we have recently found that *S. aureus* strain SA113 and its isogenic lipoprotein-deficient mutant (Δ*lgt*) did not differ much in their capacities to cause sepsis in wild-type and TLR2-deficient mice^11^. In order to elucidate if and under which conditions TLR2 may be crucial in staphylococcal infections we compared different Gram-positive pathogens including several *S. aureus* strains for their capacity to stimulate TLR2. In line with our previous finding^11^, SA113 extracellular factors in culture filtrates elicited only very weak interleukin (IL)-8 release in TLR2-transfected human embryonic kidney 293 (HEK-TLR2) cells (Fig. 1a). Similar observations were made with other Gram-positive pathogens such as enterococci, *Streptococcus pyogenes* and *Listeria monocytogenes*. When different MRSA strains were compared for their capacity to stimulate IL-8, it became obvious that hospital-associated MRSA (HA-MRSA) such as strains COL, Mu50 and N315 behaved like SA113 while highly pathogenic community-associated MRSA (CA-MRSA) such as USA300 and USA400 (ref. 30) induced secretion of approximately tenfold higher amounts of IL-8, indicating that strong TLR2 stimulation is not a constant species-specific phenomenon but differs profoundly between individual strains and may be associated with high staphylococcal virulence (Fig. 1b). Since CA-MRSA are distinguished from hospital-associated MRSA and SA113 by particularly strong activity of the global virulence regulator Agr^31^, we compared the TLR2-stimulating capacities of CA-MRSA wild-type and isogenic Agr mutants. The function or dysfunction of the Agr -system in the various strains was confirmed by quantitative real-time PCR (qRT–PCR) of the Agr-dependent RNAIII (Fig. 1i, Supplementary Fig. 1). Of note, inactivation of Agr in USA300 and USA400 resulted in drastically reduced capacities to induce IL-8 release in HEK-TLR2 cells or human polymorphonuclear neutrophil granulocytes (PMN) and tumour-necrosis factor (TNF)-α, IL-6 and IL-1β release in human peripheral blood mononuclear cells (PBMC), which demonstrated that the capacity of *S. aureus* to stimulate TLR2 depends heavily on an active Agr system (Fig. 1b-f, Supplementary Fig. 2). USA300 extracellular factors in culture filtrates stimulated HEK-TLR2 cells in a dose-dependent manner with no cytotoxic side effects at the used concentrations (Fig. 1g,h). In contrast, HEK cells not expressing TLR2 did not respond to USA300 culture filtrates confirming that the Agr-dependent IL-8 release in HEK-TLR2 cells depends on TLR2 (Supplementary Fig. 3c).

### PSM peptides are necessary for *S. aureus* TLR2 stimulation

**Figure 2:**
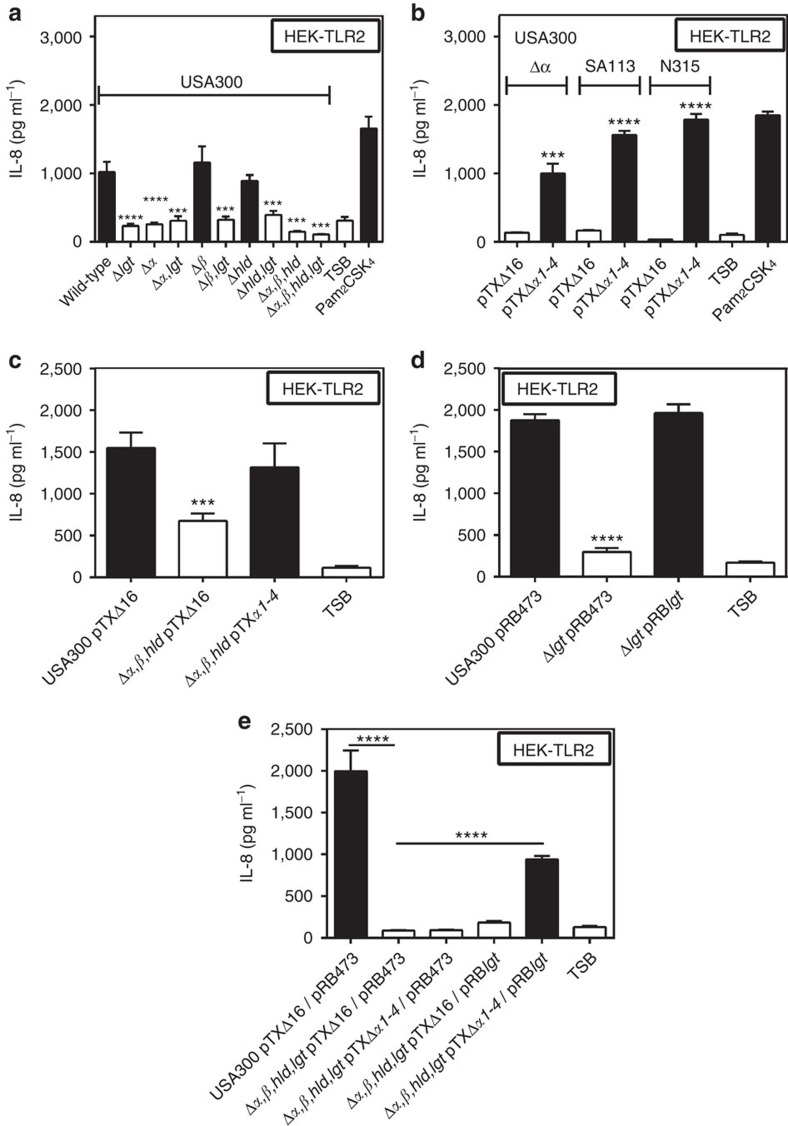
Agr-controlled PSMα peptides and lipopeptides are required for strong TLR2 activity. Inactivation of PSMα genes in *S. aureus* USA300 has a similar impact on IL-8 induction in HEK-TLR2 cells as deletion of the lipoprotein diacylglyceryl transferase gene (*lgt*) (**a**) and this phenotype can be complemented by recombinant expression of the PSMα cluster (**b**). Stimulation of HEK-TLR2 with live *S. aureus* leads to decreased IL-8 induction by the PSM mutant compared with USA300 wild-type, which can be restored by PSMα expression (**c**). The capacity of *S. aureus* Δ*lgt* to stimulate IL-8 release in HEK-TLR2 cells can be restored by transformation with an *lgt*-expressing plasmid (**d**). A USA300 mutant lacking PSMs and Lgt requires complementation with both, PSM genes and *lgt* to regain TLR2-stimulating activity (**e**). The synthetic lipopeptide Pam_2_CSK_4_ and TSB medium were used as positive and negative controls, respectively. Data represent means +/− s.e.m. of at least three independent experiments. ***P<0.001; ****P<0.0001 significantly different versus USA300 wild-type (**a**,**c**,**d**), pTXΔ*α1-4* versus empty plasmid (**b**), or as indicated (**e**) as calculated by Student's t test.

**Figure 3:**
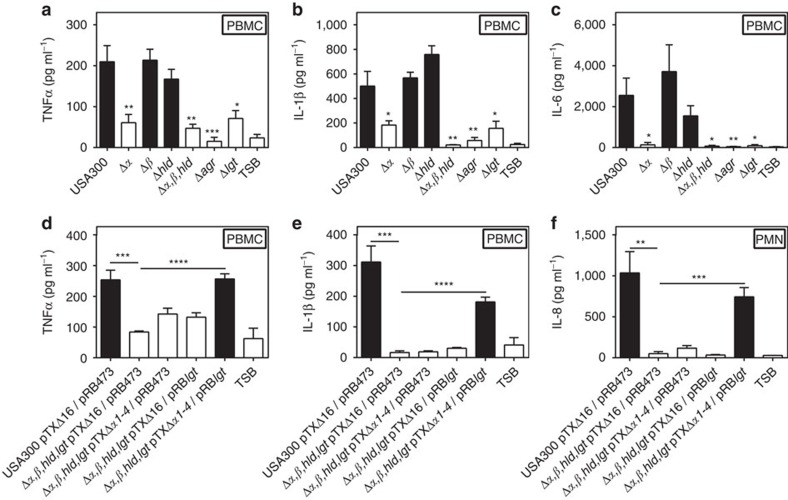
Proinflammatory cytokine induction in human PMNs and PBMCs by *S. aureus* depends on PSMα and lipopeptide expression. Absence of PSMα or *lgt* genes abrogates the capacity of *S. aureus* to stimulate TNF-α, IL-6 or IL-1β (**a**–**c**) and a USA300 mutant lacking PSMs plus Lgt requires complementation with both, PSM genes and *lgt* to regain the capacity to stimulate the release of these cytokines in human PBMCs (**d**–**e**) or IL-8 by human PMNs (**f**). Data represent means +/− s.e.m. of at least three independent experiments. *P<0.05, **P<0.01; ***P<0.001; ****P<0.0001; significantly different versus USA300 wild-type (**a**–**c**) or as indicated (**d**–**f**) as calculated by Student's *t*-test.

**Figure 4:**
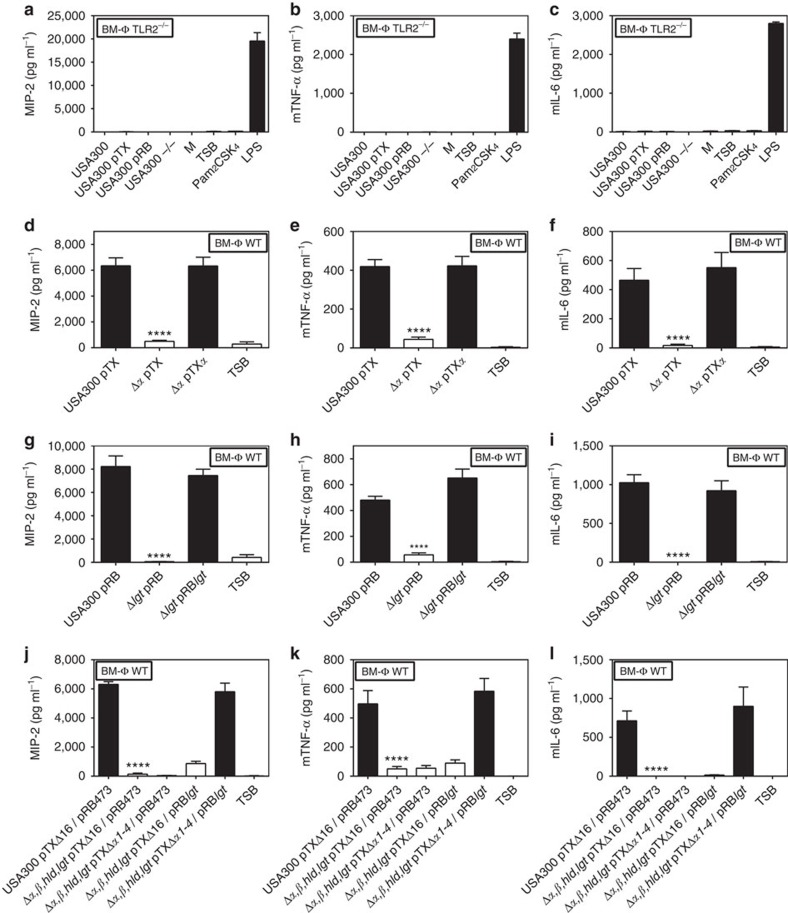
TLR2 activation in mouse bone marrow-derived macrophages (BM-ϕ) by S*. aureus* depends on expression of lipopeptides and PSMα. BM-ϕ from TLR2^−/−^ mice do not secrete MIP-2, mTNFα or mIL-6 in response to USA300 or the synthetic lipopeptide Pam_2_CSK_4_ but respond to LPS stimulation (**a**–**c**). Expression of both, PSMα and lipopeptides, is necessary to induce MIP-2, mTNFα and mIL-6 in BM- ϕ derived from wild-type mice (**d**–**l**). Data represent means +/− s.e.m. of at least three independent experiments. ****P<0.0001, significantly different versus USA300 wild-type (**d**–**l**) as calculated by Student's *t*-test.

Because Agr has a particular impact on expression of pro-inflammatory PSM peptides^25^ the impact of PSM genes on the TLR2-stimulating capacity of *S. aureus* was analysed. A USA300 mutant lacking each of the three PSM gene clusters encoding PSMα, PSMβ, and δ-toxin/Hld peptides^32^ behaved like Agr or *lgt* mutants with hardly detectable capacities to induce IL-8. PSMα peptides, the most cytolytic PSMs^24^, had the most pronounced impact on TLR2 activation, whereas inactivation of PSMβ peptides or δ-toxin did not affect the response of HEK-TLR2 to *S. aureus*. (Fig. 2a). Recombinant expression of the four PSMα peptides in Agr-deficient *S. aureus* strains such as SA113 and N315 or the PSMα-deficient USA300 Δ*α* mutant led to markedly increased IL-8 induction in HEK-TLR2 cells confirming that PSMα peptide expression is crucial for TLR2 stimulation by *S. aureus* culture filtrates (Fig. 2b). Complementation of PSM- and lipopeptide-deficient mutants with plasmid-encoded copies of the deleted genes showed that both are required for TLR2-stimulating activity (Fig. 2c–e). Similar observations were made with IL-8, TNF-α, IL-6 and IL-1β induction in human PMNs and PBMCs, with MIP-2, mTNFα or mIL-6 induction in mouse macrophages, or with IL-8 induction in HEK-TLR1/2 or HEK-TLR2/6, indicating that Agr-regulated PSMs may have essential roles in TLR2-dependent *S. aureus* cytokine induction and inflammation (Figs 3 and 4, Supplementary Fig. 3).

### PSM peptides mobilize lipopeptides to stimulate TLR2

**Figure 5:**
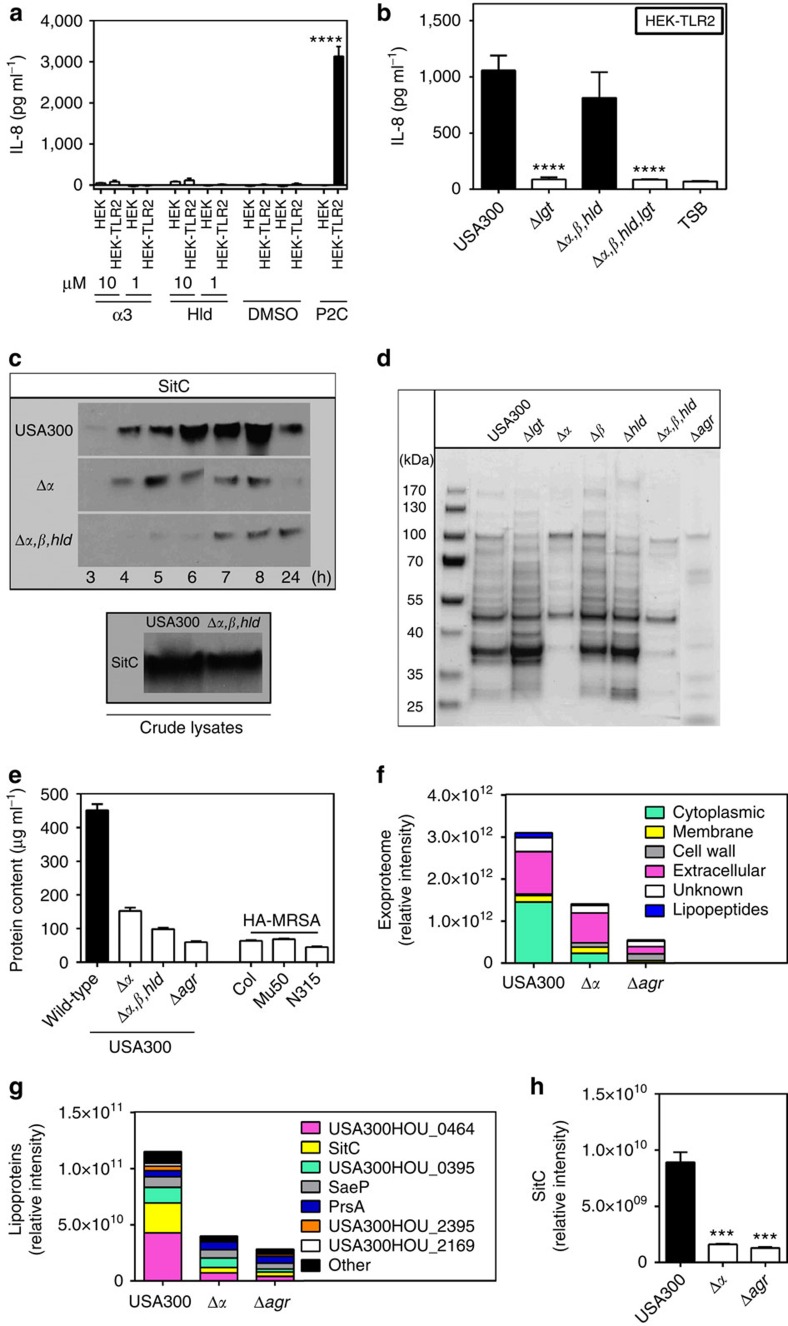
PSMα peptides do not activate TLR2 directly but are required for the release of lipoproteins from *S. aureus*. Synthetic PSMs do not stimulate IL-8 secretion by untransfected or TLR2-transfected HEK cells (**a**). PSMs do not affect the capacity of *S. aureus* USA300 cell lysates to induce IL-8 in HEK-TLR2 (**b**) but are required for efficient release of the major lipoprotein SitC with a C-terminal His tag as shown by western blotting of equal amounts of culture filtrates of USA300 wild-type, Δ*α*, and Δ*α,β,hld* (**c**, Supplementary Fig. 7). Inactivation of Agr or absence of PSMs leads to drastically altered Coomassie Blue-stained exoprotein patterns and reduced protein content in culture filtrates (**d**,**e**). Amounts of cytoplasmic proteins are also strongly reduced in USA300 PSM and Agr mutant exoproteomes compared with the wild-type strain (**f**). In contrast, signal peptide-bearing secreted proteins are not affected by deletion of PSMα peptides but the amount of this group of proteins is reduced in culture filtrates of the Agr mutant, probably because Agr controls expression of many major extracellular proteins. Overall amounts of most *S. aureus* lipoproteins and the lipoprotein SitC are reduced in culture filtrates of Agr and PSM mutants (**g**,**h**). Data in (**a**), (**b**) and (**f**) represent means +/− s.e.m. of at least three independent experiments. ***P<0.001, ****P<0.0001, significantly different for HEK versus HEK-TLR2 (**a**) and mutants versus USA300 wild-type (**b**,**f**) as calculated by Student's *t* test.

Since TLR2 stimulation is thought to be caused predominantly by bacterial lipoproteins it was analysed how PSMs and lipoproteins may play together in the induction of proinflammatory responses. The TLR2-activating capacity of Agr-positive *S. aureus* was fully dependent on lipoprotein MAMPs because inactivation of the lipoprotein-diacylglyceryl transferase gene (*lgt*) completely abrogated cytokine induction in human PBMCs, mouse macrophages, HEK-TLR2, HEK-TLR1/2 and HEK-TLR2/6 (Fig. 3, Fig. 4g–l, Fig. 2a,d, Supplementary Fig. 3). Synthetic PSMs did not stimulate HEK-TLR2 indicating that PSMs are no TLR2 agonists but may have essential roles in the expression, stability or release of lipoproteins (Fig. 5a). Of note, PSMs were only necessary for IL-8 induction when bacterial extracellular factors were used. In contrast, cell lysates of PSM-deficient USA300 mutants were equally active as those of USA300 wild-type (Fig. 5b) suggesting that even PSM mutants contain substantial amounts of TLR2 ligands but do not release them. In order to evaluate this assumption the major *S. aureus* lipoprotein SitC^19^,^33^ was quantified in USA300 wild-type and PSM mutants by western blotting. Of note, similar amounts of SitC were found cell-associated in wild-type and mutant strain while the amounts were drastically reduced in culture supernatants of the PSM mutant (Fig. 5c). Moreover, the entire exoprotein profile was substantially altered and particularly smaller protein bands corresponding to the predicted mass of many lipoproteins (∼20–40 kDa, Supplementary Data 1) were largely absent in the PSM and Agr mutants (Fig. 5d–f). Detailed label-free proteome analysis confirmed that the amounts of most lipoproteins were markedly reduced in culture filtrates of the PSMα and Agr mutants compared with the wild-type (Fig. 5g,h). All these data provided evidence for a crucial role of PSMs, which are known to have surfactant-like properties^26^, in the release of membrane-embedded lipoproteins to the extracellular space.

**Figure 6:**
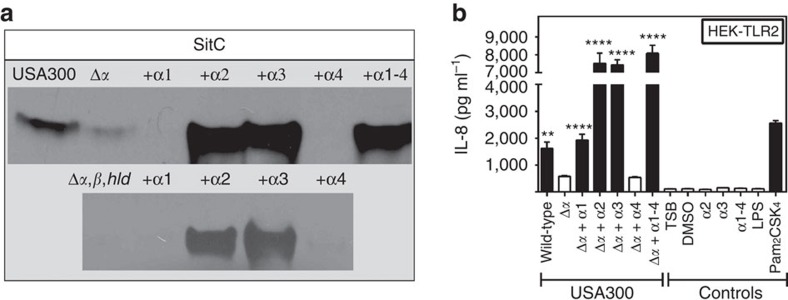
Lipoproteins can be released from *S. aureus* by incubation with synthetic PSMα peptides. Incubation of *S. aureus* USA300 PSM mutants with synthetic PSMα peptides leads to a strong increase in free His-tagged SitC detectable in western blots of culture filtrates (**a**, Supplementary Fig. 7) and restores the capacity of PSM mutant culture filtrates to stimulate IL-8 production in HEK-TLR2 cells (**b**). The synthetic lipopeptide Pam_2_CSK_4_ and TSB medium or (dimethylsulphoxide) DMSO at concentrations used to dissolve synthetic PSMs were used as positive and negative controls, respectively. Data in (**b**) represent means +/− s.e.m. of at least three independent experiments. **P<0.01; ****P<0.0001, significantly different versus USA300 Δ*α* (**b**) as calculated by Student's *t* test.

PSM-producing staphylococci are not inhibited by PSMs because they express the PSM-exporting Pmt efflux pump^34^. Nevertheless, it is tempting to assume that PSMs alter the properties of the staphylococcal cytoplasmic membrane in a way that promotes the release of lipoproteins. In order to study if exogenous PSMs can also mobilize lipoproteins *S. aureus* PSM mutants were incubated with synthetic PSMs. Indeed, supernatants of PSMα-treated PSM mutants contained strongly increased levels of SitC and exhibited potent TLR2-stimulating activity (Fig. 6a,b). In line with the assumption that PSMs may partially permeabilize the cytoplasmic membrane many cytoplasmic proteins were also much more abundant in culture filtrates of PSMα-expressing *S. aureus* compared with Agr or PSM-deficient mutants, whereas the levels of signal peptides-bearing secreted proteins remained largely unaltered in the wild-type compared with the PSM mutant. In contrast, the Agr mutant produced less extracellular proteins than the PSM mutant because many secreted proteins are Agr-regulated^34^ (Fig. 5f, Supplementary Data 1). Major toxins such as Hla, Pvl and LukAB had no impact on the overall protein amount and had no major impact on TLR2-dependent cytokine release (Supplementary Fig. 4). Nevertheless, PSM-producing *S. aureus* were not affected in their growth behaviour, viability or microscopic appearance (Supplementary Fig. 5), which confirms previous results^24^,^34^ and indicates that the PSM-mediated modulation of the cytoplasmic membrane has no detrimental impact on *S. aureus* cellular integrity.

### TLR2 is crucial for protection against PSM-producing *S. aureus*

**Figure 7:**
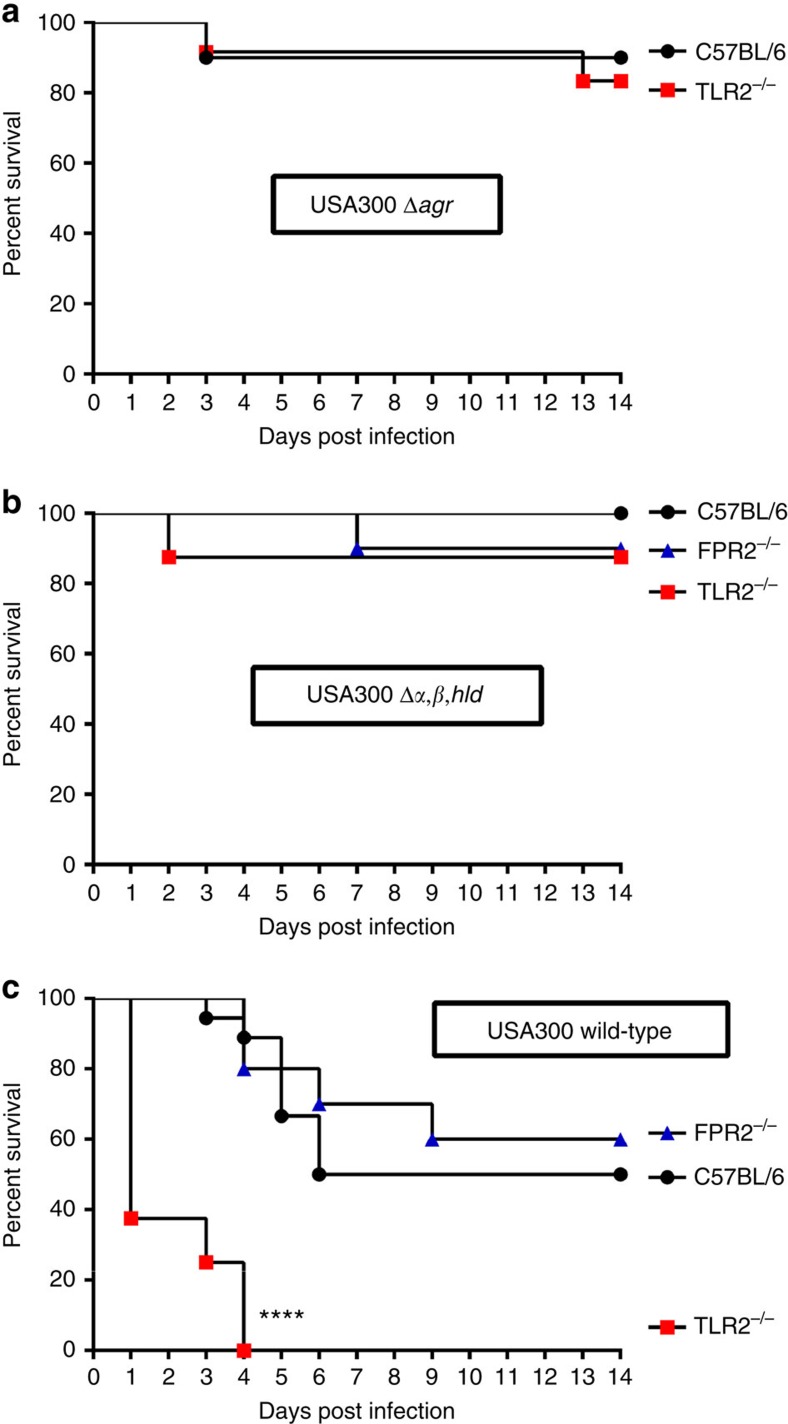
TLR2 protects mice against sepsis caused by PSM-producing *S. aureus*. TLR2 has only a minor impact on survival of mice infected with Agr or PSM-deficient *S. aureus* USA300 (**a**,**b**) but protects mice infected with USA300 wild-type (**c**). The FPR2 receptor has no major impact on the course of systemic *S. aureus* infection. Mice were infected with 1 × 10^7^ CFU of USA300 wild-type (18 non-transgenic, 8 TLR2^−/−^, 10 FPR2^−/−^ C57BL/6 animals), with USA300 Δ*α,β,hld* (8 non-transgenic, 8 TLR2^−/−^, 10 FPR2^−/−^ C57BL/6 animals), or USA300 Δ*agr* (10 non-transgenic, 12 TLR2^−/−^ C57BL/6 animals). Survival curves were monitored for statistical significance versus wild-type mice by the log-rank test.

**Figure 8:**
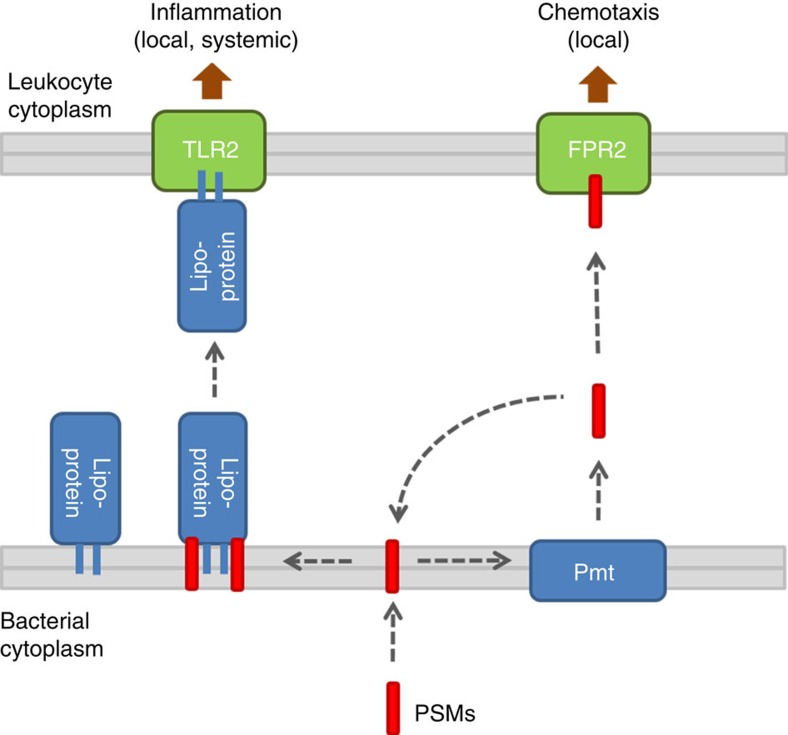
Proposed mechanism of lipoprotein release by PSMs. PSM lipopeptides cause general inflammation via TLR2 (left) while PSM gradients lead to local recruitment of leukocytes via FPR2 (right)^28^.

The finding that PSMs are crucial for TLR2-dependent cytokine induction in cell culture raised the question if PSMs contribute to TLR2 activation *in vivo*. Intravenous injection of C57BL/6 mice with *S. aureus* USA300 led to induction of proinflammatory cytokines and USA300 wild-type caused stronger inflammation than the PSM mutant (Supplementary Fig. 6). USA300 PSM or Agr mutants had lethal consequence only in a small minority of mice and mortality was similar in wild-type and TLR2-deficient mice, indicating that TLR2 does not contribute much to clearing systemic infection caused by PSM-deficient *S. aureus* (Fig. 7a,b). In contrast, USA300 wild-type was more virulent than PSM or Agr mutants, most probably as a consequence of PSM- and Agr-dependent cytotoxicity. Moreover, inactivation of TLR2 led to increased mortality in USA300 wild-type infected mice with no surviving animals 4 days after infection (Fig. 7c). Thus, TLR2 has a vital role in protection against infections caused by *S. aureus* that produce PSMs (Fig. 8).

The FPR2 receptor can sense PSM peptides and elicit neutrophil chemotaxis^27^,^28^. FPR2-deficient mice were included in the infection model to assess if FPR2 may contribute to PSM-dependent aggravation of sepsis, but wild-type and FPR2-deficient mice did not differ in sepsis. This indicates that FPR2 may be important for leukocyte recruitment in local infection as reported previously^27^ but has no major impact on systemic infection. Taken together, these data confirm that TLR2 is important in *S. aureus* infections but its capacity to contribute to host defense depends on the mobilization of lipoproteins by PSMs.

## Discussion

Our study demonstrates that the capacity of pattern recognition receptors to respond to MAMPs does not only depend on MAMP production but also on mechanisms mobilizing them. These may be human molecules such as lipopolysaccharide-binding protein and CD14 for lipopolysaccharide (LPS) release and TLR4 stimulation^35^ or, as in the case of PSMs, bacterial molecules. PSMs are also crucial toxins disrupting eukaryotic membranes at very high concentrations^24^,^26^. They may be indispensable for highly pathogenic *S. aureus*, which may use PSMs for coping with immune cells infiltrating upon strong activation of innate immunity via TLR2 or other pathways. It is interesting to note that PSMα2 and PSMα3, the most cytotoxic PSMs^24^, also had the most pronounced capacity to release lipoproteins from *S. aureus*. Thus, disrupting human cell membranes and promoting the release of bacterial lipoproteins may depend on the same surfactant-like properties of PSMs.

PSM peptides have previously been reported to have some TLR2-stimulating activity^36^, but this study has used PSMs purified from staphylococcal culture supernatants, which may have been contaminated by residual lipoproteins. In contrast, we demonstrate that synthetic PSMs have no such activity. Only staphylococci produce PSMs and high amounts are found in highly pathogenic strains such as CA-MRSA^26^,^29^. Accordingly, TLR2 may have a major role in defense against highly pathogenic *S. aureus* and only a minor role against opportunistic pathogens. This notion is in agreement with the low capacities of enterococci and *L. monocytogenes* to stimulate HEK-TLR2. *S. aureus* strains causing chronic infections such as osteomyelitis often acquire Agr mutations^37^ probably as a means to avoid strong TLR2-dependent inflammation. Many *S. aureus* strains produce the SSL3 protein, a potent inhibitor of TLR2 (refs 38, 39), which may have similar consequences as the mutation of Agr albeit without affecting the activity of virulence factors other than TLR2 agonists and PSMs.

Modulation of inflammation is a crucial strategy to treat bacterial infections, either by dampening overwhelming inflammation, for example, in sepsis or by stimulating host defense in chronic infections, which often fail to initiate an appropriate immune response^40^,^41^. *S. aureus* sepsis is always associated with exuberant, systemic inflammation^14^,^15^ and protective immune-modulatory intervention strategies are urgently needed. Our study will be important for assessing in which types of Gram-positive infections immunomodulatory interventions should be helpful. In addition to directly targeting TLR2, new types of drugs may help to either block PSM release to prevent exuberant inflammation or support lipoprotein release in chronic infections. Along this line the *S. aureus* PSM exporter Pmt has been recognized as an attractive antibiotic target^34^. Moreover, agents with surfactant-like properties such as membrane-damaging antibiotics and disinfectants may help to mobilize TLR2 ligands in infections caused, for example, by *S. aureus* Agr mutants. PSMs are only found in staphylococci but the concept of MAMP release by surfactant–like molecules may be relevant also for other pathogens, for example, by the rhamnolipids of *Pseudomonas aeruginosa*^42^. Modulating the expression or treatment with surfactant-like agents may open new avenues for therapeutic interventions.

## Methods

### Bacterial cultivation and preparation of crude lysates

Bacterial strains (Supplementary Table 1) were maintained on sheep-blood tryptic soy agar plates. Haemolysis and RNAIII expression were monitored to confirm functional Agr systems and toxin production in *S. aureus*. All bacteria were grown in tryptic soy broth (TSB, Gram-positives) or lysogeny broth (*E. coli*) supplemented with appropriate antibiotics (Supplementary Table 2) if necessary in flasks on a shaker at 37 °C. In order to induce SitC expression in *S. aureus* USA300 pTXSitC-His, bacteria were cultivated in TSB without glucose containing 0.5% xylose. Bacterial culture supernatants were obtained by centrifugation of overnight cultures and filtered through 0.2-μm pore size filters. All steps were performed at 4 °C or on ice. Twenty-four-well plates were used for small culture volumes of bacteria when adding synthetic PSM peptides to release lipoproteins. Bacterial lysates were performed using the FastPrep-24 cell disintegrator (MP Biomedicals). To this end 500 μl glass beads (150–212 μm, Sigma) were mixed with 500 μl heat-inactivated (70 °C, 30 min) bacteria (OD_600_=28) in lysis buffer (20 mM Tris/HCl, 0.1 M NaCl, pH 8). FastPrep speed was set to 5 and bacteria were lysed by three runs for 30 s. To determine the colony forming units (CFU)-to- optical density relation of bacterial strains TSB was inoculated with overnight cultures at an OD_600_ of 0.1 and the ODs were monitored at hourly intervals until the stationary growth phase was reached (growth curves). The CFU was determined with optical density adjusted overnight cultures by plating on TSB plates. To determine the ratio between live and dead cells the LIVE/DEAD BacLight Bacterial Viability Kit (Molecular Probes) was used as described by the manufacturer's instructions.

### RNA isolation

For qRT–PCR 25-ml cultures were inoculated with a preculture to an OD_600_ of 0.05 and grown for 6 h. Cells were immediately harvested and RNA stabilized by addition of 2 × vol. RNAprotect bacteria reagent (Quiagen). After centrifugation the supernatant was removed, and the pellet was quickly frozen with liquid N_2_.Then the pellets were resuspended in 1 ml TRIZOL solution (Invitrogen—Life Technologies Corporation, Darmstadt, Germany) and lysed with 0.3 ml zirconia-silica beads (Karl Roth GmbH, Karlsruhe, Germany; 0.1-mm diameter) in a high-speed benchtop homogenizer (FastPrep-24, MP Biomedicals, Germany). The lysed samples were mixed with 200 μl chloroform followed by centrifugation for 15 min at 4 °C and 15 000 r.p.m. The clear layer was transferred to a new tube and mixed with 500 μl isopropanol. After a further centrifugation and two washing steps with ethanol the RNA was carefully resuspended in RNAse-free water. To get rid of DNA contamination 5 μg ml^−1^ of each sample was mixed with 2 μl DNAse I (DNase Treatment and Removal kit, Ambion, Life Technologies) and 1 μl recombinant RNasin ribonuclease inhibitor (Promega, Madison, WI, USA) and incubated for 30 min at room temperature. Then DNAse was inactivated by adding inactivation reagent (DNase Treatment and Removal kit, Ambion, Life Technologies and RNA concentration was analysed.

### qRT–PCR

For relative quantification standards of the gyrase gene *gyrB* and for RNAIII were generated from pooled RNA with the primers for *gyrB* forward: 5′-CAAATGATCACAGCATTTGGTACAG-3′ and reverse: 5′-CGGCATCAGTCATAATGACGAT-3′ or RNAIII forward: 5′-GTGATGGAAAATAGTTGATGAGTTGTTT-3′ and reverse: 5′-GAATTTGTTCACTGTGTCGATAATCC-3′, respectively, as described in ref. 31. qRT–PCR was performed using the QuantStudio 3 instrument (Applied Biosystems, Thermo Fisher Scientific) in combination with the Power SYBR Green RNA-to-C_T_ 1 Step Kit (Applied Biosystems). Master mixes were prepared according to the manufacturer's instructions.

### Stimulation of cell lines

HEK cells stably transfected with the human TLR2, TLR1/2, TLR2/6 genes or un-transfected HEK cells were purchased from Invivogen. HEK-TLR2 cells were cultivated in 75-cm^2^ culture flasks using 20 ml of growth medium (Dulbecco's modified eagle medium (DMEM), 10% fetal calf serum (FCS), 100 μg ml^−1^ normocin, and 10 μg ml^−1^blasticidin). Un-transfected HEK cells were cultivated in DMEM, 10% FCS, 20 mM L-glutamine, and 1,000 u ml^−1^penicillin/streptomycin. Cells were seeded into 24-well cell culture plates and cultivated until confluence was reached. Growth medium was then replaced by medium without FCS containing appropriately diluted stimuli. Culture filtrates were used at 0.25% (TSB), 2% (TSB with 0.5% xylose and lacking glucose) or 5% (IMDM) final concentration according to bacterial densities reached in the various media. Diluted culture filtrates exerted no toxicity towards HEK cells as analysed with the Cytotoxicity Detection Kit (Roche Applied Sciences). No stimulatory activity was detected in non-inoculated media at corresponding dilutions. Protein concentrations of crude lysates were determined with the Bradford assay (BioRad) and 100 ng ml^−1^ per lysate were used for stimulation. For stimulation with live bacteria 1 × 10^6^ CFU *S. aureus* from overnight cultures grown in TSB were added per well and incubated with HEK cells for 18 h at 37 °C and 5% CO_2_ to allow expression and release of PSMs and lipoproteins. Subsequently bacterial growth was stopped by adding 200 μg ml^−1^ gentamicin to avoid overgrowth and cytotoxicity and incubation was continued for another 16 h. The synthetic lipopeptide Pam_2_CSK_4_ (1 ng ml^−1^ in stimulation medium) and cell culture medium with TSB concentrations corresponding to diluted culture filtrates were used as positive and negative controls, respectively. Formylated PSM peptides (PSMα1, PSMα2, PSMα3 and PSMα4) with the recently published sequences^24^ were synthesized at the Interfaculty Institute of Cell Biology (Department of Immunology, University of Tübingen). After stimulation supernatants were collected by centrifugation for 10 min at 250 × *g* and stored at −20 °C before use. Cytokines were measured using ELISA kits (R&D Systems) according to the manufacturer's instructions.

### Stimulation of primary cells

Human PMNs and PBMCs were isolated from fresh human blood of healthy volunteers by standard Ficoll/Histopaque gradient centrifugation as described recently^27^ and stimulated with diluted bacterial culture filtrates (1.5% for TSB culture filtrates) or crude bacterial lysates (100 ng ml^−1^ protein) in 96-well plates. 5 × 10^5^ PMNs or PBMCs were seeded in cell culture medium (very low endotoxin-Roswell Park Memorial Institute Medium (RPMI) 1640, 2 mM sodium pyruvate, 2 mM L-glutamine, 100 u ml^−1^ penicillin/streptomycin, 10 mM 4-(2-hydroxyethyl)-1-piperazineethanesulfonic acid (HEPES)) and incubated for 5 h at 37 °C and 5% CO_2_. Primary bone marrow-derived macrophages (BM-Φ) were generated from 12-week-old female and male C57BL6J wild-type (Harlan Laboratories) or isogenic TLR2^−/−^43 mice as described previously^44^. Briefly, femora and tibiae were flushed with cold PBS. Obtained cells were cultivated in medium (DMEM, 10% FCS, 5% human serum albumin (HSA), 1% penicillin/streptomycin, 1% glutamine, 0.5% sodium pyruvate and 20% supernatant of L929 cells producing macrophage colony-stimulating factor for differentiation) for 7 days in 15-cm uncoated cell culture dishes and subsequently resuspended in ice-cold PBS on ice. BM-ϕ were seeded at a density of 0.25 × 10^6^ per ml in 24-well plates and allowed to adhere overnight followed by stimulation with culture filtrates (2%) diluted in stimulation medium (DMEM, 10% FCS, 1% glutamine, 0.5% sodium pyruvate, 1% penicillin/streptomycin) for 24 h. Cytokines were measured as described above.

### Deletion of *lgt* in *S. aureus* USA300 LAC

For deletion of the *lgt* gene in USA300 LAC the flanking regions of the *lgt* gene were amplified by PCR using primer with added restriction sites for *Eco*RI/*Sac*I (N-terminal) and *Sac*I/*Bgl*II (C-terminal) (Supplementary Table 3). The plasmid pBASE6 was digested with *Eco*RI and *Bgl*II and ligated with the flanking regions of *lgt*. After amplification of pBASE6-*Δlgt* in *E. coli* DH5α and *S. aureus* RN4220, the plasmid was used to transform USA300 and the *lgt* gene was deleted by homologous recombination as described previously^45^.

### SitC release and detection

Bacterial cultures expressing SitC-His under xylose induction were adjusted to densities of OD_600_=0.1, supplemented with 200 μg ml^−1^ PSMα peptides and cultivated for appropriate times in 24-well plates under agitation at 37 °C. Culture filtrates were obtained by centrifugation for stimulation of HEK cells as described above or for detection of SitC-His by western blotting. The protein concentration of culture filtrates was determined by Bradford assay (BioRad) and a volume corresponding to 25 μg protein of USA300 wild-type and the same volume from mutant strains were concentrated with 10 μl Strataclean Resin beads (Agilent Technologies). After washing with 1 ml PBS, beads were resuspended in twofold loading dye (Pierce), boiled at 99 °C for 10 min, cooled down on ice, and loaded on SDS-HEPES polyacrylamide gels (12%, Gel Pierce). Ten microlitres of PageRuler (Thermo) was used as molecular weight marker. SDS-PAGE was performed according to the manufacturer's instructions (Pierce). Proteins were either visualized by Coomassie blue staining or transferred from SDS gels to a nitrocellulose membranes for western blotting. The membrane was blocked for 30 min with Pierce blocking solution and washed twice with Tris-buffered saline and Tween and one time with Tris-buffered saline. The first antibody (mouse anti-5His-IgG from QIAGEN, 0.2 mg ml^−1^ stock solution diluted 1:10,000) was applied for 60 min in blocking solution. After washing the second antibody (goat anti-maus-IgG-HRP from Merck Millipore, 0.2 mg ml^−1^ stock solution diluted 1:30,000) in Tris-buffered saline with 10% skim milk powder was added for 60 min. The membrane was then washed again. For visualization of the SitC-His bands on the membrane the western blotting luminol reagent (Santa Cruz) was applied and chemiluminescence was detected on an x-ray film.

### Transmission electron microscopy

Transmission electron microscopy analysis was performed as described earlier^34^. Briefly, bacterial cultures grown in TSB medium were harvested at late exponential growth phase (6 h). The cells were fixed overnight at 4 °C by 2.5% glutaraldehyde in 0.1 M sodium cacodylate and rinsed three times with 0.1 M sodium cacodylate. Samples were further fixed with 0.5% osmium tetroxide/0.8% potassium ferricyanide in 0.1 M sodium cacodylate and then with 1% tannic acid. The fixed samples were dehydrated by washing with ethanol and then transferred to acetone. After embedding into epon/araldite and polymerization, thin sections were cut and stained with 4% samarium acetate and Reynold's lead citrate. The sections were viewed at 80 kV on a Tecnai BT Spirit transmission electron microscope (FEI) and digital images were acquired with a Hamamatsu ORCA HR camera system (Advanced Microscopy Techniques).

### Quantitative label-free proteomics

Three biological replicates of USA300 wild-type or mutant culture filtrates grown overnight in TSB were analysed. The same protein amount measured by Bradford assay in culture filtrates of the various strains was used for exoprotein precipitation with 10% ice-cold trichloroacetic acid overnight at 4 °C. After centrifugation at 13,200 r.p.m. at 4 °C for 15 min the supernatants were discarded and precipitated proteins were air dried. For nanoliquid chromatography–mass spectrometry/mass spectrometry analysis dried proteins were dissolved in a buffer containing 6 M urea, 2 M thiourea, 10 mM Tris at pH 8.0 and digested in solution with trypsin as described previously^46^. Peptide mixtures were then separated on an EasyLC nano- high-performance liquid chromatography (Proxeon Biosystems) coupled to an Linear Trap Quadrupole (LTQ) Orbitrap Elite mass spectrometer (Thermo Fisher Scientific) as described elsewhere^46^ with the following modifications: Peptides were eluted with an 87-min segmented gradient of 5–33–90% high-performance liquid chromatography solvent B (80% acetonitrile in 0.5% acetic acid). Each sample was run in technical triplicates. Acquired mass spectrometry spectra were processed with the MaxQuant software package version 1.2.2.9 (ref. 47) with integrated Andromeda search engine^48^ as described previously^46^. Database searches were performed against a target decoy *S. aureus* USA300 database obtained from Uniprot, containing 5,295 protein entries and 248 commonly observed contaminants. The label-free algorithm was enabled as was the ‘match between runs' option^49^. Label-free quantification (LFQ) protein intensities from the MaxQuant data output were used for relative protein quantification. Different volumes of culture filtrates needed for precipitation of equal amounts of proteins were considered when calculating the original protein contents from determined LFQ intensities. Detected proteins were sorted by their subcellular localization using the software PSORTb v.3.0 (www.psort.org). The database of lipopeptide prediction PRED-LIPO was used to identify lipoproteins (*S. aureus* USA300 predicted 63: www.bioinformatics.biol.uoa.gr/PRED-LIPO-results/). Culture filtrates were compared for total protein content (∑LFQ of all proteins) and composition sorted by subcellular localization (∑LFQ of each subcellular localization). Differences of single proteins between mutants are listed in Supplementary Data 1.

### Mouse bacteremia model

TSB medium was inoculated with *S. aureus* strains from a pre-culture and grown to mid-exponential growth phase (2–3 h). Bacteria were harvested, washed and diluted in sterile PBS. CFU were determined by diluting and plating on TSB plates. Six-week old female C57BL/6 wild-type mice purchased from Harlan Laboratories, C57BL/6 TLR2^−/−^43, or C57BL/6 FPR2^−/−^50 mice were challenged with 1 × 10^7^ live bacteria in 0.2 ml PBS injected into the tail vein as described recently^43^. Survival and disease progression was monitored over 14 days. Blood was collected from some mice 6 and 17 h after infection. Cytokine/chemokine levels in blood serum were determined following the manufacturer's instructions with a custom-made Luminex assay including magnetic beads for the measurement of CXCL2 (MIP-2), IL-1β, IL-6 and TNFα (R&D Systems, Minneapolis, MN, USA). The samples were analysed on a Bio-Plex MAGPIX Multiplex Reader (Bio-Rad Laboratories, Munich, Germany) with Bio-Plex Manager MP Software (Bio-Rad) and ELISA (R&D Systems, Minneapolis, MN, USA).

### Statistics

Statistical analysis was performed using Graph Pad Prism 5.0. Unpaired two-tailed Student's *t*-test was used to compare two groups unless otherwise noted. Survival of mouse strains was compared with the log-rank test. Data represent mean and s.d. or s.e.m. as indicated.

### Ethic statements

Human PMNs and PBMCs were isolated from venous blood of healthy volunteers in accordance with protocols approved by the Institutional Review Board for Human Subjects at the University of Tübingen. Informed written consent was obtained from all volunteers. Animal experiments were performed according to German law with permission of the responsible authorities (H3/12 und H3/13, Regierungspräsidium Tübingen).

### Data availability

The mass spectrometry proteomics data have been deposited to the ProteomeXchange Consortium via the PRIDE^51^ partner repository with the dataset identifier PXD004283. These data were further processed and are provided as source data for figure 5f–h with the article (Supplementary Data 1). The authors declare that the data supporting the findings of this study are available within the article and its supplementary information files.

## Additional information

**How to cite this article:** Hanzelmann, D. *et al*. Toll-like receptor 2 activation depends on lipopeptide shedding by bacterial surfactants. *Nat. Commun.* 7:12304 doi: 10.1038/ncomms12304 (2016).

## Supplementary Material

Supplementary Information Supplementary Figures 1-7, Supplementary Tables 1-3 and Supplementary References

Supplementary Data 1 Quantitative label-free proteomics. Three biological replicates of USA300 wild-type, PSM and Agr mutant culture filtrates were analysed for protein content and composition. Label-free quantification (LFQ) protein intensities are listed for detected proteins of each culture filtrate (sheet: quantitative label-free). Proteins were sorted for their subcellular localization. ∑LFQ of each protein was compared between wild-type and mutants (sheets: cytoplasmic, membrane, cell wall, extracellular, unknown and lipopeptides). Culture filtrates were compared for composition sorted by subcellular localization (∑LFQ of each subcellular localization) (sheet: ∑LFQ).
