# Supplementary material for: Toll-like receptor 2 activation depends on lipopeptide shedding by bacterial surfactants
Source: Nat Commun. 2016 Jul 29;7:12304. doi: 10.1038/ncomms12304 (PMC4974576; doi:10.1038/ncomms12304)
Supplement: Supplementary Information — Supplementary Figures 1-7, Supplementary Tables 1-3 and Supplementary References [file ncomms12304-s1.pdf]

Supplementary Figures:

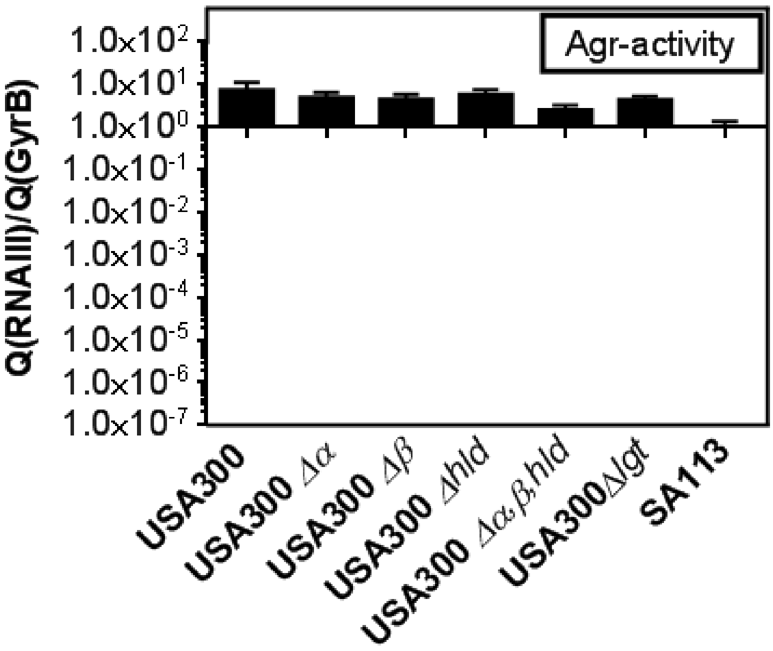

Supplementary Figure 1. Agr-activity of USA300 wild type and PSM or Lgt mutants. USA300 wild type and isogenic mutants lacking *psm* or *lgt* genes show comparable RNAIII expression levels measured by qRT-PCR after 6 h growth in TSB. Data represent means +/- SEM of at least three independent experiments.

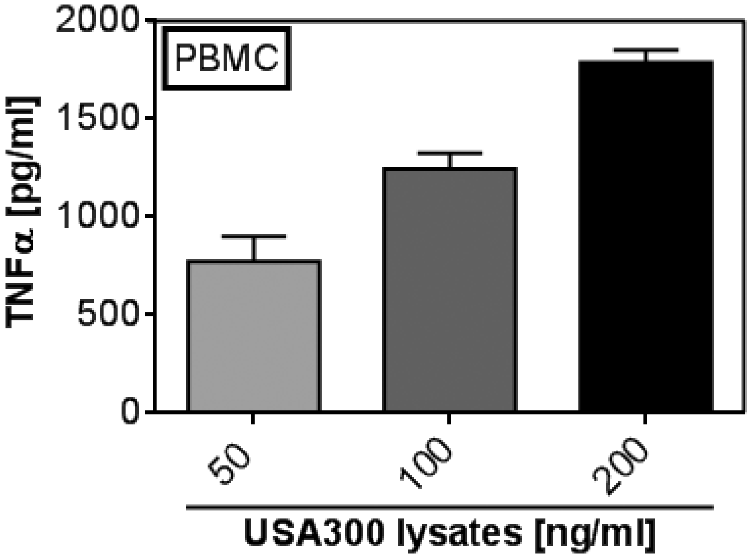

Supplementary Figure 2. USA300 whole cell lysates cause dose-dependent TNFα release in PBMCs. Bacterial lysates are less toxic compared to culture filtrates and can be used in higher concentrations leading to higher and dose-dependent TNFα release. Data represent means +/- SD of at least three independent experiments.

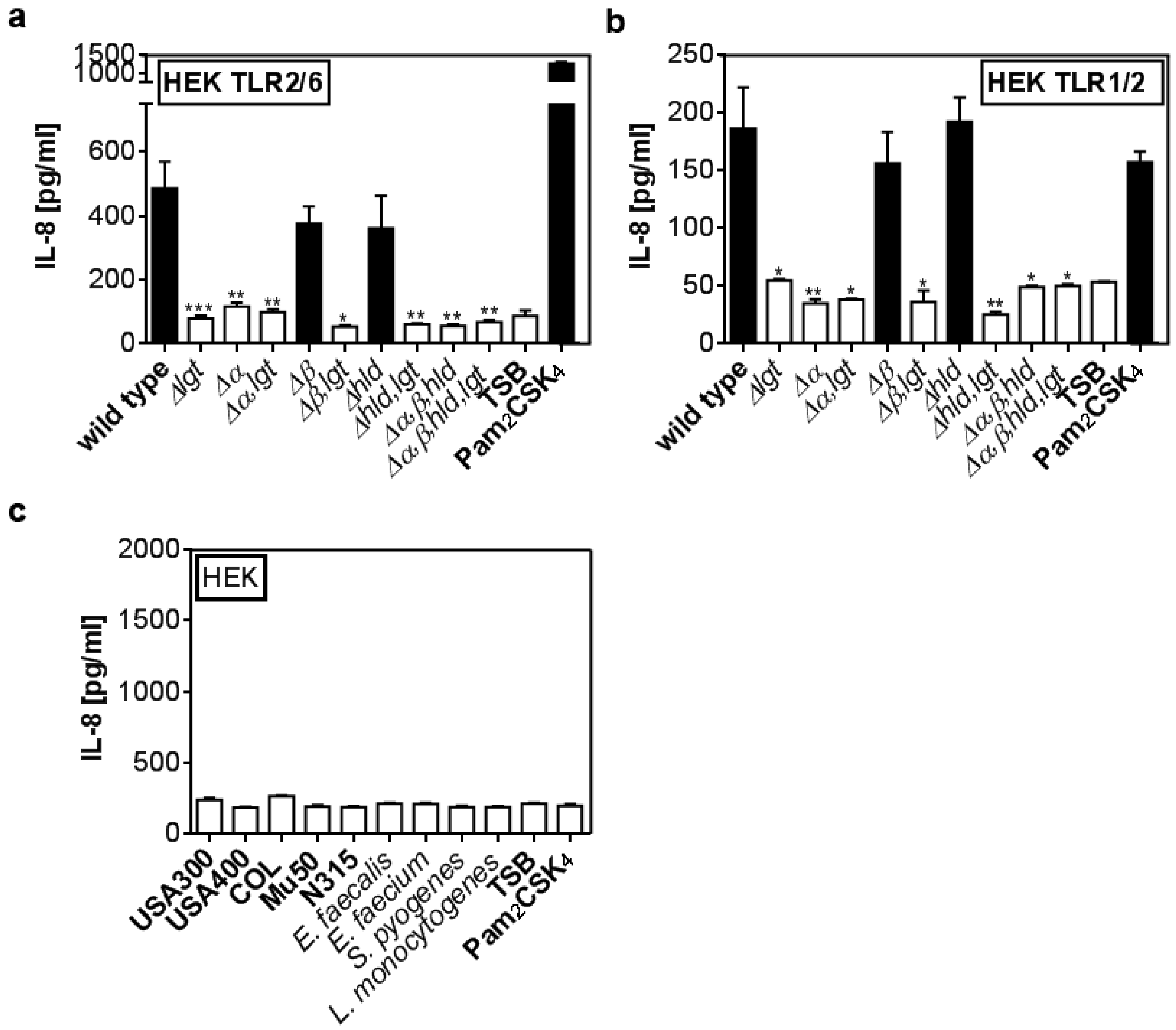

Supplementary Figure 3. IL-8 release by HEK cells depends on the presence of TLR2 and co-receptors. Gram-positive bacteria cause similar patterns of IL-8 release in TLR2/6 and TLR1/2-transfected HEK cells (**a-b**) compared to TLR2-mono-transfected HEK cells (compare Fig. 2a). Gram-positive bacteria cannot stimulate IL-8 release in un-transfected HEK cells (**c**). Data represent means +/- SEM of at least three independent experiments. *p < 0.05, **p < 0.01; ***p < 0.001, significantly different vs. USA300 wild type (**a-b**) as calculated by Student's t-test.

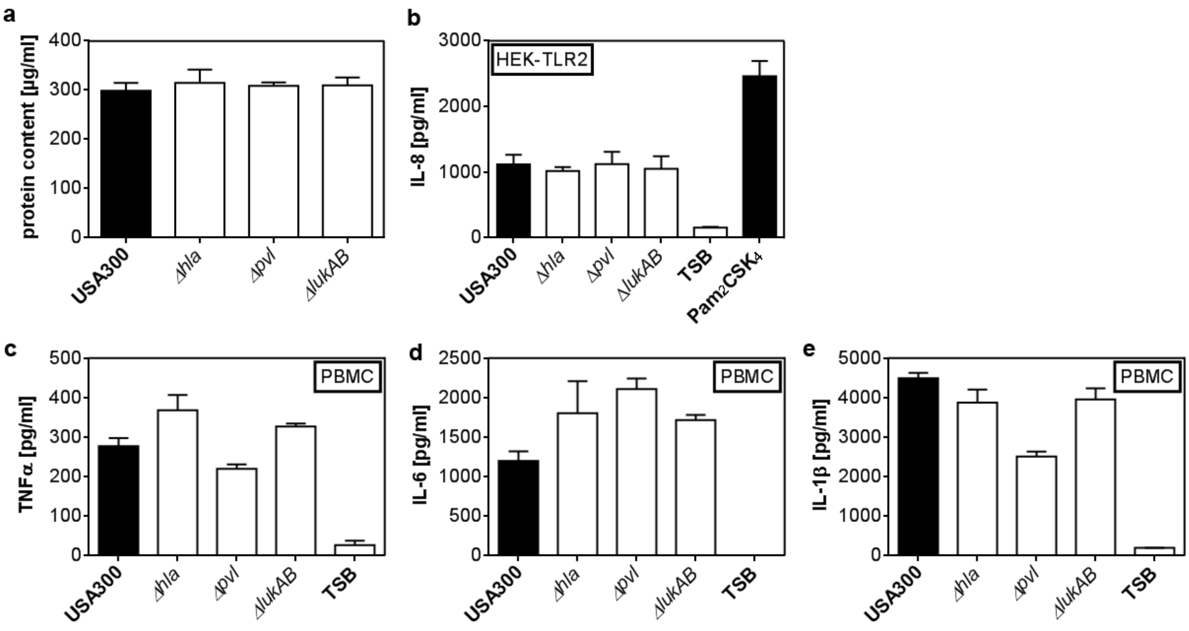

Supplementary Figure 4. Major *S. aureus* toxins do not affect overall exoprotein levels or TLR2-dependent cytokine responses. Culture filtrates of USA300 toxin mutants have the same protein amounts (**a**). Culture filtrates of USA300 wild type or toxin mutants stimulate comparable amounts of IL-8 in HEK-TLR2 cells or IL-8, TNFα, IL-6 and IL-1β in human PBMCs (**b-e**). Data represent means +/- SEM of at least three independent experiments.

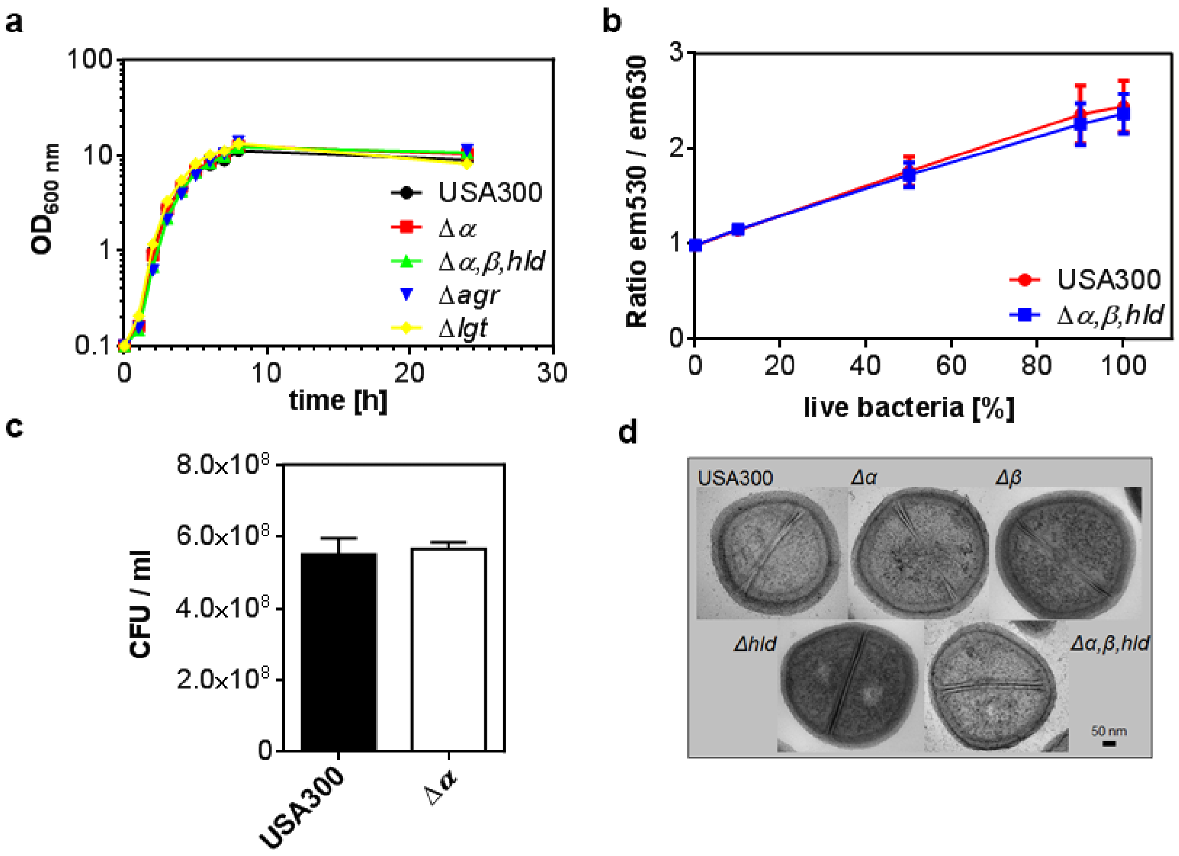

Supplementary Figure 5. Expression of PSMα peptides has no impact on growth and viability of *S. aureus* USA300. USA300 and isogenic mutants lacking PSM genes, *agr*, or *lgt* show comparable growth in TSB (**a**), equal numbers of live and dead bacteria in overnight cultures (**b**), and CFU in cultures adjusted to the same OD_{600} (**c**). Transmission electron microscopy indicates that PSM expression does not alter the integrity of the *S. aureus* cell membrane and envelope (**d**). Data represent means +/- SD of at least three independent experiments (**b-c**).

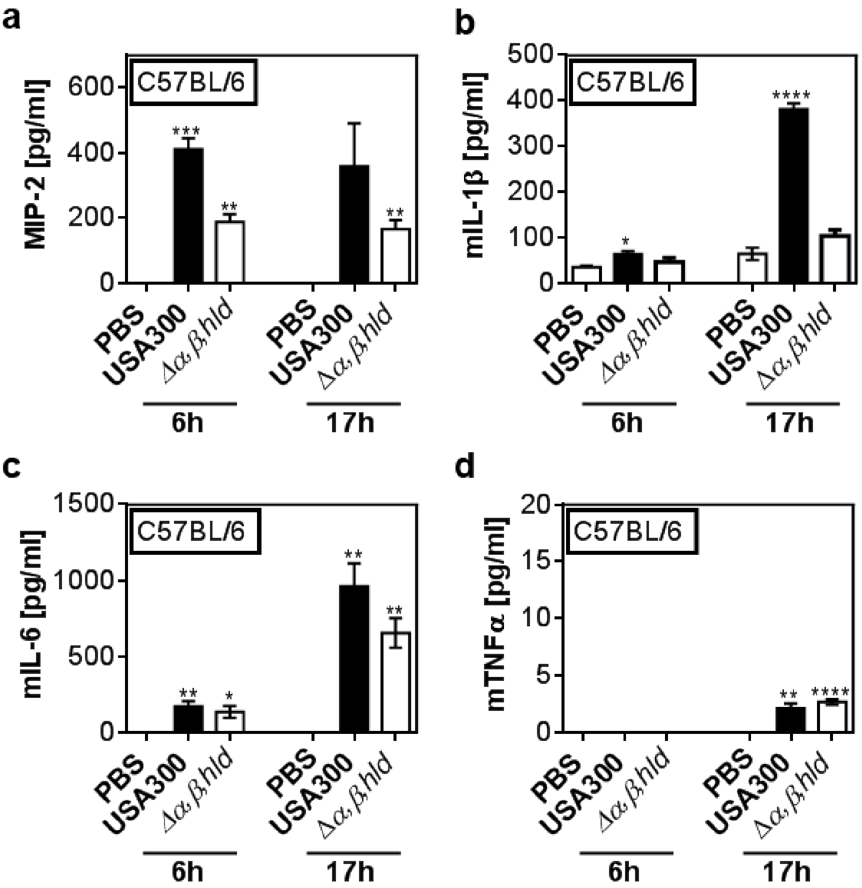

Supplementary Figure 6: Mouse Infection with *S. aureus* USA300 leads to systemic proinflammatory cytokines response. C57BL/6 mice were infected with 1 x 10^7 CFU of USA300 wild type (n=5) or USA300 Δ*α,β,hld* (n=5). Blood was collected 6 h and 17 h after infection. Cytokines in serum were measured using ELISA. Data represent means +/- SEM. *p < 0.05, **p < 0.01; ***p < 0.001; ****p < 0.0001; significantly different vs. PBS-injected animals (**a-d**) as calculated by Student's t-test.

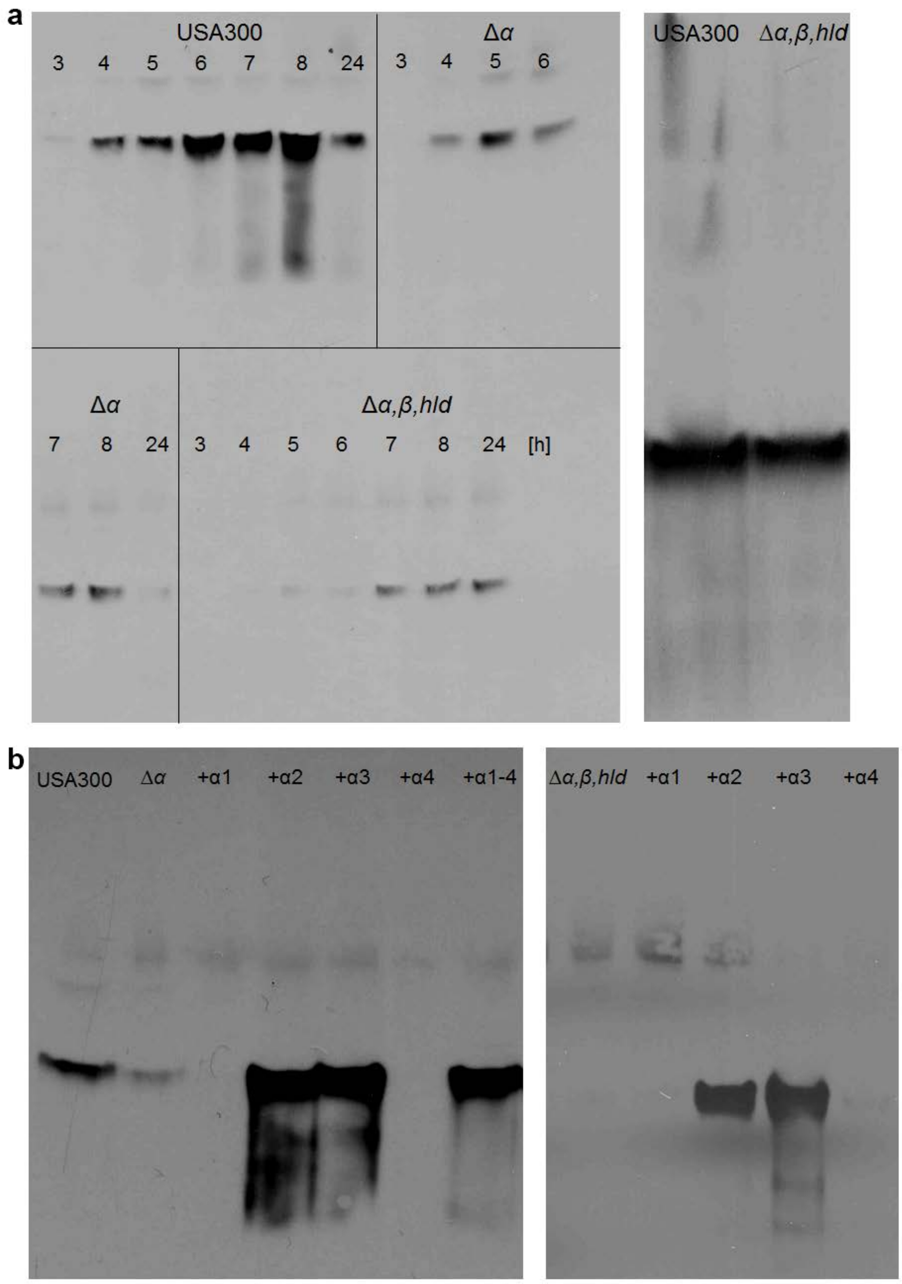

Supplementary Figure 7: Full Western blots for SitC detection in culture filtrates or lysates. Cultures of SitC-His expressing bacteria under xylose induction were collected and SitC release was monitored and compared over time. Crude lysates of USA300 and the PSM-deficient mutant were analyzed for amounts of cell-bound SitC (**a**). PSM mutants grown over night in the presence of synthetic PSMα peptides showed increased release of SitC to the culture supernatant compared to the mutants alone (**b**). Blots correspond to those shown in Figure 5c (**a**) and Figure 6a (**b**) of the main manuscript.

Supplementary Table 1: Bacterial strains and deletion mutants used in this study.

Species	Strain	Genotype	Reference
Staphylococcus aureus	USA300 LAC (CA-MRSA)	Wild type	Wang, R., et al.[1]
		Δagr	Wang, R., et al.[1]
		Δα	Wang, R., et al.[1]
		Δβ	Wang, R., et al.[1]
		Δhld	Wang, R., et al.[1]
		Δα,β,hld	Joo, H.S., et al.[2]
		Δlgt	This study
		Δα,lgt	This study
		Δβ,lgt	This study
		Δhld,lgt	This study
		Δα,β,hld,lgt	This study
		Δhla	Münzenmayer, L. [3]
		Δpvl	Münzenmayer, L. [3]
		ΔlukAB	Münzenmayer, L. [3]
	USA400 MW2 (CA-MRSA)	Wild type	Wang, R., et al.[1]
		Δagr	Wolz, C.
	USA100 Mu50 (HA-MRSA)	Wild type	Wang, R., et al.[1]

	USA100 N315 (HA-MRSA)	Wild type	Wang, R., et al.[1]
	USA500 COL (HA-MRSA)	Wild type	Wang, R., et al.[1]
	SA113 (MSSA)	Wild type	Stoll, H. et al.[4]
	RN4220 (MSSA)	Wild type	Peng, HL et al.[5]
Enterococcus faecalis	V583 (VRE)	Wild type	Bloes, D.A. et al.[6]
Enterococcus faecium	BK2241	Wild type	Bloes, D.A. et al.[6]
Streptococcus pyogenes	BK2192	Wild type	Diagnostics unit, University Hospital Tübingen
Listeria monocytogenes		Wild type	Diagnostics unit, University Hospital Tübingen
Escherichia coli	DH5α	F– Φ80*lac*ZΔM15 Δ(*lac*ZYA-*arg*F) U169 *rec*A1 *end*A1 *hsd*R17 (rK–, mK+) *pho*A *sup*E44 λ– *thi*-1 *gyr*A96 *rel*A1	Invitrogen

Supplementary Table 2: Plasmids used in this study for complementation, gene deletion, or overexpression of SitC.

Plasmid	Purpose	Resistance	Reference
pTX$_{\Delta}$16	Empty control vector	Tetracycline 12 µg/ml	Wang, R., et al.[1]
pTX$_{\Delta}$*α1-4*	Constitutive expression of *psmα1-4*	Tetracycline 12 µg/ml	Wang, R., et al.[1]
pRB473	Empty control vector	Chloramphenicol 10 µg/ml	Stoll, H. et al.[4]
pRB*lgt*	Constitutive expression of *lgt*	Chloramphenicol 10 µg/ml	Stoll, H. et al.[4]
pTXSitC-His	Xylose-inducible SitC-His expression	Tetracyclin 12 µg/ml	Stoll, H. et al.[4]
pBASE6	Empty plasmid for targeted gene deletion	Ampicillin 100 µg/ml	Geiger T. et al.[7]
pBASE6*Δlgt*	Deletion of *lgt*	Ampicillin 100 µg/ml	This study

Supplementary Table 3: Primers used for deletion of *lgt* in USA300.

Primer	Sequence
Fragment I EcoRI	GATgaAtTCGCTGGTGAAGAAGGAC
Fragment I SacI	CGTgagCTCAGTGGTCCTAAGTTAAATGCC
Fragment II SacI	CGTgagCTCAGTGGTCCTAAGTTAAATGCC
Fragment II BglII	TTGAGAtctAAGATATTGGAATAGTATTTG
pBASE6 PI sequencing	CCTCAAGCTAGAGAGTCATTACCCC
pBASE6 PII sequencing	CTACTTCTTTCAAACTCTCTCTACG

Supplementary References

1. Wang, R. *et al.* Identification of novel cytolytic peptides as key virulence determinants for community-associated MRSA. *Nat Med* **13**, 1510-1514, doi:10.1038/nm1656 (2007).
2. Joo, H. S., Cheung, G. Y. & Otto, M. Antimicrobial activity of community-associated methicillin-resistant Staphylococcus aureus is caused by phenol-soluble modulin derivatives. *J Biol Chem* **286**, 8933-8940, doi:10.1074/jbc.M111.221382 (2011).
3. Munzenmayer, L. *et al.* Influence of Sae and Agr regulated factors on the escape of Staphylococcus aureus from human macrophages. *Cell Microbiol*, doi:10.1111/cmi.12577 (2016).
4. Stoll, H., Dengjel, J., Nerz, C. & Gotz, F. Staphylococcus aureus deficient in lipidation of prelipoproteins is attenuated in growth and immune activation. *Infection and immunity* **73**, 2411-2423, doi:10.1128/IAI.73.4.2411-2423.2005 (2005).
5. Peng, H. L., Novick, R. P., Kreiswirth, B., Kornblum, J. & Schlievert, P. Cloning, characterization, and sequencing of an accessory gene regulator (agr) in Staphylococcus aureus. *J Bacteriol* **170**, 4365-4372 (1988).
6. Bloes, D. A., Otto, M., Peschel, A. & Kretschmer, D. Enterococcus faecium stimulates human neutrophils via the formyl-peptide receptor 2. *PLoS One* **7**, e39910, doi:10.1371/journal.pone.0039910 (2012).
7. Geiger, T. *et al.* The stringent response of Staphylococcus aureus and its impact on survival after phagocytosis through the induction of intracellular PSMs expression. *PLoS Pathog* **8**, e1003016, doi:10.1371/journal.ppat.1003016 (2012).
